# Advances in Soft and Dry Electrodes for Wearable Health Monitoring Devices

**DOI:** 10.3390/mi13040629

**Published:** 2022-04-16

**Authors:** Hyeonseok Kim, Eugene Kim, Chanyeong Choi, Woon-Hong Yeo

**Affiliations:** 1 Georgia Institute of Technology, George W. Woodruff School of Mechanical Engineering, Atlanta, GA 30332, USA; hyeonseok.kim@me.gatech.edu (H.K.); ekim399@gatech.edu (E.K.); cchoi90@gatech.edu (C.C.); 2 IEN Center for Human-Centric Interfaces and Engineering, Institute for Electronics and Nanotechnology, Georgia Institute of Technology, Atlanta, GA 30332, USA; 3 Wallace H. Coulter Department of Biomedical Engineering, Georgia Institute of Technology and Emory University, Atlanta, GA 30332, USA; 4 Parker H. Petit Institute for Bioengineering and Biosciences, Neural Engineering Center, Institute for Materials, Institute for Robotics and Intelligent Machines, Georgia Institute of Technology, Atlanta, GA 30332, USA

**Keywords:** soft–dry electrode, wearable device, health monitoring, physiology signal sensing, manufacturing

## Abstract

Electrophysiology signals are crucial health status indicators as they are related to all human activities. Current demands for mobile healthcare have driven considerable interest in developing skin-mounted electrodes for health monitoring. Silver-Silver chloride-based (Ag-/AgCl) wet electrodes, commonly used in conventional clinical practice, provide excellent signal quality, but cannot monitor long-term signals due to gel evaporation and skin irritation. Therefore, the focus has shifted to developing dry electrodes that can operate without gels and extra adhesives. Compared to conventional wet electrodes, dry ones offer various advantages in terms of ease of use, long-term stability, and biocompatibility. This review outlines a systematic summary of the latest research on high-performance soft and dry electrodes. In addition, we summarize recent developments in soft materials, biocompatible materials, manufacturing methods, strategies to promote physical adhesion, methods for higher breathability, and their applications in wearable biomedical devices. Finally, we discuss the developmental challenges and advantages of various dry electrodes, while suggesting research directions for future studies.

## 1. Introduction

The activities of human organs and tissues are driven by changes in electrical potentials called electrophysiological (EP) signals [1]. The major signals include electrocardiography (ECG) [2,3], electroencephalography (EEG) [4,5], electromyography (EMG) [6], and electrooculography (EOG) [7] signals. This can reflect the physiological state of the human body and is a critical indicator for evaluating various physiological activities of the body in clinical treatment, improvement of physical condition, behavioral analysis, etc. [1]. Among them, the ECG signal directly reflects the health state of the heart and is used as a measure to detect cardiovascular diseases such as arrhythmias and valve abnormalities [8]. EEG signals can provide important medical information about brain diseases such as dementia, tumors, and epilepsy [9,10]. EOG signals can monitor eye movement status for sleep monitoring or feedback of retinal stimulation, paving the way for mobile eye therapy and eye movement control [7]. EMG signals are closely related to muscle disease and fatigue, and have recently been widely used in gesture recognition, and prosthetic control [11]. Therefore, it is crucial when develop systems that continuously monitor human bioelectrical signals to detect potential health problems and ensure patient health and safety. According to the materials, EP signals are captured by bio-signal electrodes, including wet and dry electrodes. Silver/Silver chloride (Ag/AgCl) electrodes, a traditional wet electrode, have been widely used to acquire EP signals due to their low skin-to-electrode impedance, low cost, and ease of use. Still, some drawbacks limit their use in long-term monitoring and wearable health monitoring applications [12]. In particular, gels used in wet electrodes can potentially cause skin irritation to the patient. They can dehydrate and coagulate after prolonged use, which can increase signal detection noise and degrade signal quality [13]. With the recent advent of nano/micro-processes and material-based stretch/flexible electronic devices, bio-signal electrodes have made significant progress over the past few years. In particular, dry electrodes that do not utilize wet gels have become a promising alternative through continuous optimization, as they achieve improvements in signal quality, ease of use, and portability [14]. Based on this, integration with circuit and wireless communication systems forms a device of a new independent wireless platform, demonstrating remarkable research achievements in wearable applications for human-machine interfaces and solutions for health monitoring at the clinical level [7,8].

**Figure 1:**
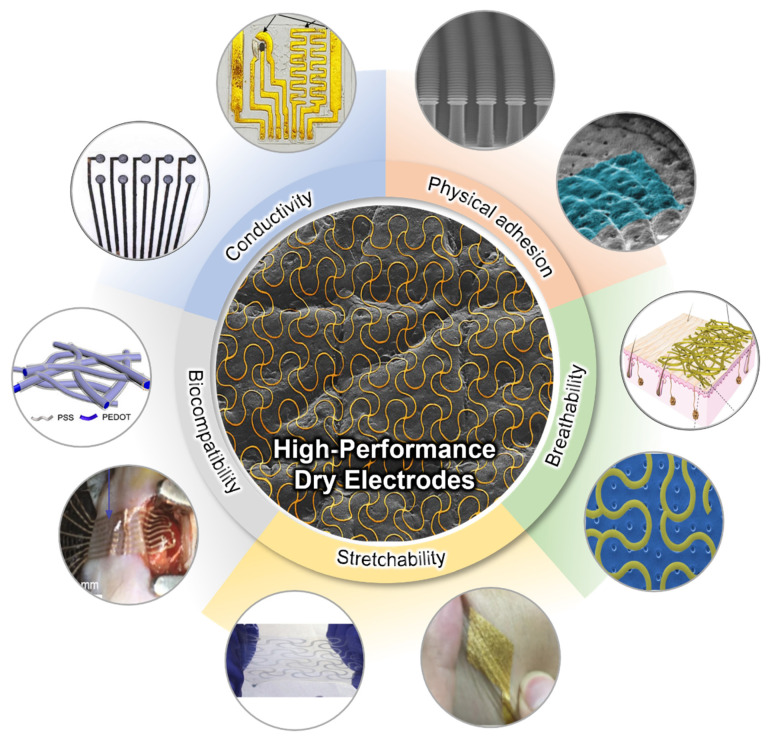
Schematic illustration of the requirements for high-performance dry electrodes. (Reprinted with permission from *Sci. Rep.* **2021**, *11*, 14823, Copyright 2021, Scientific Reports), (Reprinted with permission from *Sci. Transl. Med.* 2021, *13*, 612, Copyright 2021, Science Translational Medicine), (Reprinted with permission from *ACS Mater. Lett.* 2021, *3*, 9, 1385–1393, Copyright 2021, ACS), (Reprinted with permission from *Nat. Commun.* 2021, *12*, 3710, Copyright 2021, Nature Communications), (Reprinted with permission from *Sci. Adv.* 2017, *3*, 3, Copyright 2017, AAAS), (Reprinted with permission from *Nat. Commun.* 2021, *12*, 4731, Copyright 2018, Nature Communications), (Reprinted with permission from *Nat. Biomed. Eng.* 2019, *3*, 194–205, Copyright 2019 Nature communications), (Reprinted with permission from *Nat. Nanotechnol.* 2017, *12*, 907–913, Copyright 2017 Nature Nanotechnology), (Reprinted with permission from *Adv. Sci.* 2018, *5*, 1700771, Copyright 2018, Wiley), (Reprinted with permission from *ACS Appl. Mater. Interfaces* 2019, *11* (18), 16951–16957, Copyright 2019 ACS), (Reprinted with permission from *Adv. Mat.* 2013, *25* (20), 2773–2778, Copyright 2013 Wiley).

High-quality signal acquisition is critical for these applications. EP signals typically exhibit a small signal amplitude, less than 5 mV [15]. In addition to this, its low signal-to-noise ratio (SNR) causes randomly unstable signal changes from external or internal stimuli, and is highly susceptible to such interference [16]. Therefore, to collect high-quality and stable EP signals, a high-performance dry electrode that maintains reliable mechanical/electrical performance is needed. Figure 1 depicts the requirements for the high-performance soft electrode for wearable physiology monitoring. The electrode with high conductivity enhances the SNR in EP signal measurements due to its high surface electric displacement field and high charge carrier density [17]. While conventional bulk metal electrodes have excellent conductivity, they do not have flexibility/stretchability for wearable applications. This soft feature of the electrode is an essential factor in maintaining electrical performance as a bio-signal electrode, despite mechanical deformation such as bending, stretching, and torsion resulting from human movement. To analyze these features, the electrode’s properties are evaluated through various on-skin sensing devices as well as repeated bending/stretching tests [18]. Due to the human skin deforming up to 50% when moved, proper stretchability must be guaranteed for stable signal detection [19]. The physical adhesion of the electrode affects signal acquisition reliability. In particular, the conformal contact between the skin interface and the electrode is immensely related to improving signal quality [20]. The adhesive performance capable of sufficiently adhering to the skin provides high signal quality by reducing the motion artifact and enhancing long-term usability. In addition, electrodes use materials with high biocompatibility, such as carbon-based materials (CNT and graphene), metals (gold and platinum), and polymers (PEDOR:PSS and PPy) [21]. They can prevent health problems due to skin irritation or cytotoxicity during the long-term monitoring of bio-signals, which is an essential element as a high-performance dry electrode [22]. Humans generate more than 40 g/h/m^2^ of water from the skin every day [23]. Therefore, the breathability of the electrode is important for the user’s comfort and wearability, and for preventing skin problems during long-term monitoring. In addition, since moisture remaining between the electrode and the skin creates a signal drift, proper ventilation significantly impacts EP signal quality [24].

This review provides an overview of recent research achievements in soft–dry electrodes for wearable health monitoring applications. Section 2 explores various material-based dry electrodes, their manufacturing process, performance evaluation, and their application to bioelectrodes. Section 3 summarizes multiple approaches and applied research to improve physical adhesion, including adhesive and conformal contact. In Section 4, we provide an overview of various methods to enhance the breathability of bioelectrodes. Finally, we summarize research achievements on high-performance soft–dry electrodes and suggest perspectives for wearable healthcare applications.

## 2. Dry Electrodes with Various Materials

### 2.1. Significance of Material Selection in Dry Electrode Fabrication

Recent research in biosignal electrodes proposes a variety of materials suitable for soft, dry electrodes with high signal quality. Key material characteristics determining usability in dry electrodes are divided into electromechanical properties, including conductivity, stretchability, and biocompatibility. Commercial Ag/AgCl wet electrodes achieve low skin–electrode impedance through gel skin preparation in advance of electrode attachment, maximizing the skin-to-electrode contact area, and the gel solution provides additional conductivity [25]. Skin-to-electrode impedance proves to be one of the most challenging properties to be assessed in designing dry electrodes, due to the number of influencing variables, such as skin-to-electrode contact area and electrode material type. High-impedance electrodes also appear to be more susceptible to low-frequency noise, especially during warm and humid recording environments anticipated during long-term signal monitoring by dry electrodes [26]. Intrinsic material properties highly influence high-performance dry electrode characteristics such as flexibility and biocompatibility. For example, elasticity and microstructural porosity are critical properties associated with the long-term measurability of biosignals from wearable electronics [27]. Carbon and metal nanomaterials have been traditionally popular conductive materials that are suitable for printed electronics [28]. Recent advancements in material science also introduce conductive polymer compounds along with carbon and metal nanomaterials to be biocompatible, as outlined by usage in skin regeneration treatments [29]. Minimal cell toxicity is key when designing electrodes intended for clinical use to achieve long-term and reliable biopotential signals under robust conditions. Cytotoxicity is a key biomedical indicator to prevent cell death caused by disruptions such as cell membrane damage, protein synthesis prevention, irreversible receptor binding, etc. [30]. Recent studies further highlight the importance of assuring the biocompatibility of materials, since requirements broadly vary according to multiple variables such as the sensor–skin contact method, exposure time, and material degradation over time [31]. Electrode-related skin abrasion refers to all types of skin lesions caused by electrode placements for biosignal monitoring [32], which must be addressed in long-term measurement for patient safety and comfortability. Revolutions in manufacturing technologies now allow for novel manufacturing processes, such as inkjet printing, laser cutting, photolithography, and electrospinning, applicable to dry electrode research. These methods allow for the processing of various electrode-based materials with high precision and customizability [27]. Soft, flexible, and conductive electrode materials featured in this section will demonstrate high-quality signal monitoring, emphasizing high skin formality and conductivity through varying material selection and property optimization by fabrication methodologies.

### 2.2. Metal-Based Electrodes

**Figure 2:**
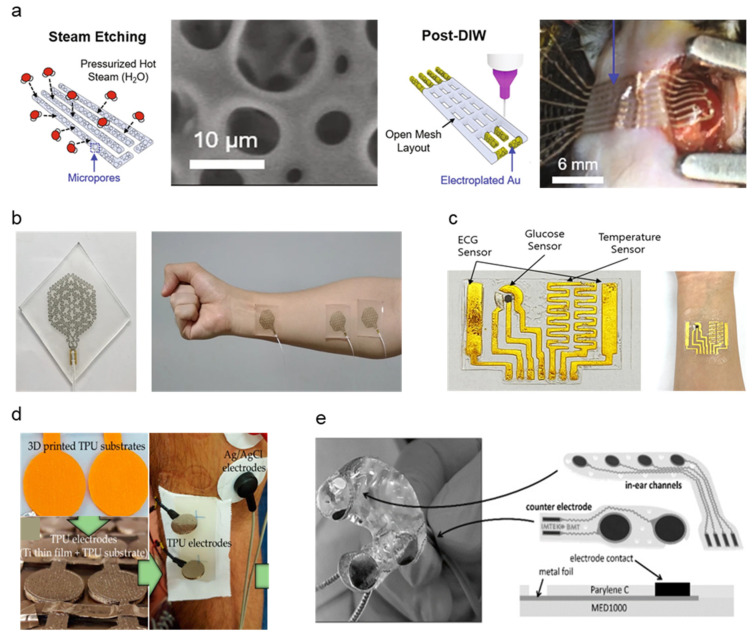
Schematic diagrams and images of metal-based dry electrodes: (**a**) Ag-Au electrode fabrication and microporous structure demonstration on the epicardial surface of the murine heart (Reprinted with permission from *Nat. Commun.* 2021, *12*, 3710, Copyright 2021, Nature Communications) [41]. (**b**) Photograph of Ag-PTFE electrode-based EMG sensor implemented on the forearm (Reprinted with permission from *NPG Asia Mater.* 2021, *13*, 4, Copyright 2021, NPG Asia Materials) [42]. (**c**) Au-layered PDMS patch-type ECG sensor conformed around wrist (Reprinted with permission from *Sci. Rep.* 2021, *11*, 14823, Copyright 2021, Scientific Reports) [43]. (**d**) Titanium-thin film electrode fabrication onto TPU substrate and sEMG measurement demonstration. (Reprinted with permission from *Materials* 2020 *13* (9), 2135, Copyright 2020, MDPI) [45]. (**e**) Multi-layer structure of assembled Ear-EEG electrode composite of platinum, silver, and MP35N metal sheets (Reprinted with permission from *Sensor* 2021, (11), 3176 (2020), Copyright 2020, MDPI) [46].

Metal, also utilized in traditional gel Ag/AgCl electrode form factors [33], has been a widely popular choice for electrode fabrication due to its high conductivity, low skin–electrode impedance, and ease of manufacturability. Ag, particularly in nanowire and ink-deposited form, is preferable due to the allowance of various fabrication methods and embedding in flexible polymer substrates for maximized flexibility [34]. Although Ag is considered widely biocompatible and safe for skin, based on comparative studies with common metals used as in-body implants such as stainless steel (SS) [35,36], skin irritation due to Ag is a common issue among patients and should be assessed for flexible dry electrode fabrications to meet long-term monitoring standards. Studies evaluated in this review aim to mitigate such issues by investigating metal fabrication methods and a combination of cost-effective but less biocompatible metals such as Ag and copper (Cu) integrated with polymer substrates or highly biocompatible noble metals such as gold (Au), titanium (Ti), and platinum (Pt) [37,38,39,40]. Table 1 summarizes the characteristics of soft–dry electrodes. Despite Ag posing inflammatory risks and having the potential for cytotoxicity, research has demonstrated the safety of usage in low dosages while taking advantage of its physicochemical properties and biological functionality, such as high antimicrobial efficiency [39]. A soft, sponge-like biosensor developed by Kim et al. implements a flexible substrate to achieve high stretchability and strain resistance [41]. Ag flakes are steam-etched into the porous silicone surface area, maximizing electrode contact area with the skin surface. A significant increase in electrode-skin contact area from the microporous nature of the electrode material contributes to low impedances at 2.1, 1.5, and 1.0 kΩ. Impedance data were measured using electrochemical references using a pH solution resembling human blood properties (pH 7.2 at 23 °C) at 40, 150, and 1000 Hz ECG signals. A water-soluble medical tape was added to ensure easy integration across the epicardial placement surface (Figure 2a). The addition of the stiffening layer significantly reduced minimum adhesion energy through strong capillary adhesion to the epicardial surface. The rapid custom prototyping of electrodes based on printable sponge-like foam substrates allowed the custom application to epicardial ECG by applying ultrasound-generated 3D models to transfer onto custom-patterned mesh sensors with conductive layers directly. Thin Au sheets are electroplated over the Ag flake layer to promote biocompatibility. The in vivo compatibility and anti-biofouling properties of the custom print electrode were addressed by assessing cell toxicity, proving that the Au coating over Ag flakes significantly increased cell variability. Such findings support non-toxicity during in vivo applications. However, a granuloma formation on the epicardial surface was observed for 14 days of post-implant of the device, suggesting the need for further design improvements for long-term epicardial monitoring.

**Table 1:** Summary of metal-based soft–dry electrodes.

Electrode Material	Manufacturing Method	Conductivity/ Resistivity	SNR/Contact Impedance	Stretchability	Thickness	Ref.
Ag flake, Au	3D Direct ink write, Steam etching, Metallization, Electroplating	1.0 kΩ (@ 40, 150, 1000 Hz)	2.1, 1.5, 1.0 kΩ (@ 40, 150, 1000 Hz)	>100%	≥50 μm	[41]
Ag-PTFE	Magnetron sputtering system	3.09–17.23 Ω/sq	N/A	40%	20 nm	[42]
Au, PDMS	Thermal release tape transfer, Laser patterning, MPTMS treatment, Spin coating, Stencil masking	10–150 Ω	N/A	<50%	250 μm	[43]
Au nanoparticle	Electrodeposition	270 mΩ/sq	ECG 51% EMG 63%	N/A	N/A	[44]
Ti thin film	Magnetron sputtering with glancing/oblique angle deposition (GLAD/OAD)	1–15 × 10^−6^ Ωm	SS: 10 kΩ TPU: 200–250 kΩ	N/A	1–1.5 mm	[45]
Pt thin film with Cu co-deposition	Electrodeposition	N/A	<5 kΩ @ 20 Hz	N/A	5–20 μm	[46]

Gold nanoparticles (AuNP) have been applied to various biomedical research, such as cancer-targeting agents owing to material stability, biofunctionability, and biocompatibility [47]. A semitransparent, stretchable hybrid Ag-polytetrafluorethylene (Ag-PTFE) on polyurethane (PU) substrate electrode was developed by Yoon et al., showing excellent sheet resistance (3.09–17.23 Ω/sq) and strain resistance (0–40%) [42]. The hybrid material consists of the PTFE as a controlling matrix for Ag particles’ agglomeration and stretchability improvement. The Ag and PTFE conductors are sputtered onto a PU substrate by a magnetron sputtering system (deposition thickness: 20 nm). A laser patterned EMG sensor was fabricated by co-sputtering the Ag-PTFE onto a silicone substrate spin-coated with solution-based polyimide (PI), which later separated the patterned electrode from the silicone-PI substrate via dipping in hot water (90 °C) and transferred to a glass substrate. Compared to traditional photolithography, the laser patterning method provides a direct patterning method that is comparatively time and cost-effective with ease of manufacturability. The final EMG sensor was packaged with adhesive and stretchable polydimethylsiloxane (PDMS) polymer from the top for patient application (Figure 2b). The surface characteristics of the resulting EMG sensor demonstrate high water resistance due to the strong interaction between carbon and fluorine particles of the PTFE, forming an incomplete wetting surface, which makes it an excellent hydrophobic material for self-cleaning dry electrodes. Water resistance was measured by contact angles of two solutions with known surface energy components, measuring 64.53°, which is considered applicable in wearable electronics. While microelectromechanical system (MEMS) technologies are widely used in flexible electronics fabrication, they are often expensive due to processes that require costly equipment (e.g., vacuum deposition, photolithography, wet/dry etching). Kim et al. propose a gold-based electrode fabricated by plotter cutter machines and surface treatment with trimethoxysilane (MPTEMS) solution before transferring the Au electrode onto a PDMS substrate (Figure 2c) [43]. The cutter-based fabrication process is more cost-effective than MEMS metallization and is also applicable in metal formation and selective encapsulation. MPTEMS treatment of the gold electrode further increases adhesion with the PDMS layer, increasing the fabrication reliability of thin-width gold electrodes up to stable fabrication at 0.2 mm minimum gold line width. The high stretchability of PDMS polymers enables the Au-PDMS electrode to stretch up to 50% while maintaining low resistance during cyclic strain testing (~100 Ω). The electrode’s electrophysiological sensing abilities were confirmed via ECG signal measurements from both phantom simulators and human subjects.

Yun et al. developed a noble-metal skin enhanced electrode by surface modification using Au in film sheets and nanoparticle states [44]. The Enlarged Surface-Area Flexible Skin Electrode (ESASE) proposed achieves performance improvement through electrodeposition, maximizing the skin-to-electrode contact area. While Au was selected to be the conductive metal material for the electrode, an electroplating methodology was used to maximize surface area. A 100 nm gold thin film was deposited to a highly flexible and skin conformal PI substrate. Au-nanoparticles were electroplated onto the gold film surface. Electrodeposition increased the surface area of the electrode by 1.5 times compared to the bare gold film electrodes, reducing interference impedance and thermal noise, thus significantly reducing signal-noise ratio (SNR). The fabricated electrode was compared against bare Au film electrodes and commercial Ag/AgCl gel electrodes using ECG and EMG measurements. In comparison to bare Au, the ESASE improved SNR by 51% for ECG and 63% for EMG. ESASE SNR values were also on par with Ag/AgCl reference electrodes, demonstrating performance exercising current gold standards. The ESASE notably outperformed reference electrodes during motion artifact analysis using pseudo-impulse response and demonstrated a 95% reduction in motion noise than Ag/AgCl electrodes.

Titanium is an alternative biocompatible noble metal widely used in clinical implants and is notable for its low corrosivity and lightweight properties [48]. A Ti-thin film electrode was compared on stainless steel (SS) and thermoplastic polyurethane (TPU) substrates for clinical sEMG measurements by S. Rodrigues et al. [45]. (Figure 2d) Ti films are fabricated by physical vapor deposition (PVD), particularly the magnetron sputtering with glancing/oblique angle deposition (GLAD/OAD) technique, are highly variable and tunable for adjusting conventional columnar metal thin films at various deposition angles. Sputtering incidence angles varied from 0–80°, which resulted in the varying microstructures. An increase in depositional angle showed an increase in electrode resistivity caused by a subsequent increase in mechanical porosity of the material, as measured in the resistivity of thin films deposited at 80°, which was 15 times higher than thin films with 0° incidence angle. 60° incidence angle was proved to be a good balance between high microstructural stability and electrical behavior. The fabricated Ti-TPU and Ti-SS electrodes were assessed against reference Ag/AgCl electrodes. Despite positive feedback on the usability and simplicity of both Ti-TPU and Ti-SS electrodes by clinical subjects and operators, TPU electrodes showed a lack of electrode-wire connection stability caused by the insulating nature of the TPU polymer. The increased sensor impedance leads the TPU electrode to be at a higher risk of noise and motion artifacts. On the other hand, the SS electrode showed high SNR in the 20.7 dB range comparable to Ag/AgCl SNR in the 22.8 dB range. Both TPU and SS electrodes demonstrate better usability and biocompatibility, as electrodes were reusable and allowed for multiple sEMG recordings without replacement. Despite the low electrical conductivity of Ti, Ti and Ti-alloys exhibit high specific strength and high biocompatibility due to the formation of a thin oxide layer which enables high corrosion resistance [49]. Ti-thin film electrodes by the S. Rodrigues’ group demonstrate the sufficiency of Ti as dry electrode base material despite low conductivity. Pt holds favorable noble metal properties such as lower ductility than Au and resistance against native oxide layer formation, which lowers conductivity as seen in Ti [46]. Comparable to the methodology adopted by the Yun group, a highly porous in-ear EEG measurement system developed applies electrodeposition processes to create porous platinum surfaces with Cu co-deposition Cu was selected as a favored co-deposition material due to a redox potential higher than noble metals and lower than hydrogen. The iterative electroplating method of Pt and Cu enables surface enlargement, increasing the active skin–electrode contact surface. Fabricated electrode arrays are assembled onto acrylic resin hard shells cast from personalized ear impressions (Figure 2e). Fifty iterations of fabrication obtained 5–20 μm low-thickness Pt electrodes. An additional 10 μm topside coding using Perylene-C was added to encapsulate the exposed Pt electrode area to improve skin contact. While motion artifacts were mitigated by otoplasty casting, further motion noise due to sweat drifting remains as untested variables. Despite such favorable Pt characteristics, the high cost of Pt due to scarcity and applicability [50] proposes a challenge for large-scale manufacturing of cost-effective Pt dry electrodes.

### 2.3. Carbon-Based Electrodes

Interest in low-dimensional carbon nanomaterial application in the biological field has aroused interest for the past 30 years. Carbon nanotube (CNT) and graphene, each discovered in 1991 and 2004, sensationalized the biomedical industry from drug delivery to electronics due to their small particle size, large surface area, and conductive and optical properties [51]. Carbon black (CB), although quite dated, has recently been rediscovered to be a favorable candidate for bio-nanomaterial due to excellent electrical conductivity, dispersibility in solvents, facile fabrication, and fast electron transfer kinetics, not to mention cost-effectiveness [52]. This section will outline various low-dimensional carbon nanomaterials for dry electrode applications fabricated in conjunction with property-enhancing additives and flexible substrates. Table 2 summarizes carbon-based electrodes. Multidimensional carbon nanomaterials (e.g., CNT, graphene, CB) presented in this section have demonstrated biocompatibility in various bio application conditions [53,54].

**Figure 3:**
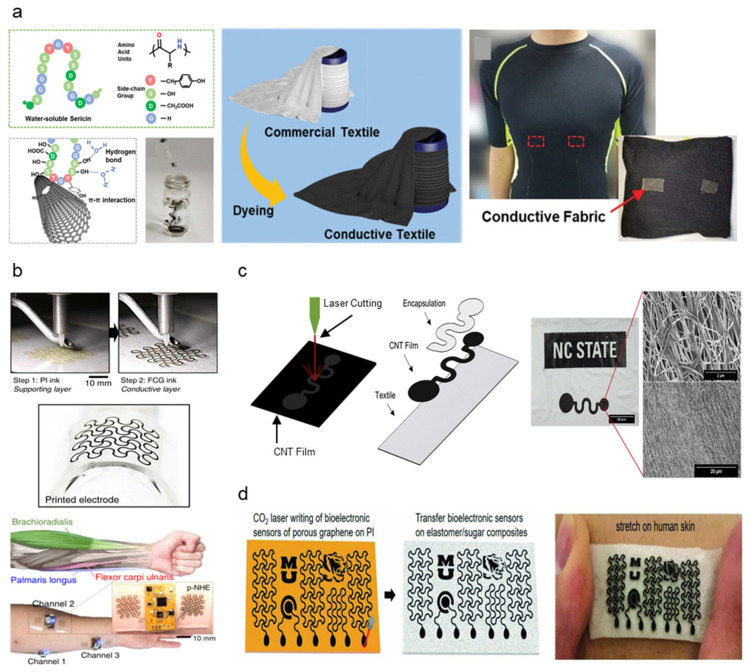
Carbon-based dry electrode fabrication and demonstration: (**a**) Chemical composition of SSCNT ink and fabrication procedure into conductive textiles (Reprinted with permission from *Adv. Mater.* 2020, *32*, 2000165, Copyright 2020, Wiley) [55]. (**b**) Fabrication steps of PI and graphene ink onto stretchable nanomembrane surface and close-up image of the printed electrode (Reprinted with permission from *Nat Commun.* 2020 *11*, 3450, Copyright 2020, Nature Communications) [56]. (**c**) CNT electrode fabrication process via laser cutting and encapsulation, optical image of electrode microstructure transferred to the textile substrate (Reprinted with permission from *Carbon* 2020, *168*, 673–683, Copyright 2020, Elsevier) [57]. (**d**) Porous LIG-based electrode fabrication on the soft substrate by laser writing and skin stretchability demonstration (Reprinted with permission from *Adv. Mater.* 2018, *30*, 1804327, Copyright 2018, Wiley) [58].

Carbon nanotube (CNT) is a common carbon nanomaterial favored for biosensing applications due to its electrical and mechanical properties along with high stability [60]. Carbon-based materials have demonstrated promise in biopotential sensing based on attractive properties, such as large contact areas and high electrical conductivity [61]. CNT-based flexible sensors are also verified to be easily printable using the direct-ink writing, which shows promise in the facile manufacturing of flexible, conductive dry electrodes [62]. However, CNT possessed biocompatibility issues from cellular uptake and cytotoxicity [63]. Liang et al. incorporated sericin proteins originating from silk biomaterials as stabilizing and biocompatible agents in CNT inks [55]. Sericin proteins provide good biocompatibility, adjustable gelation properties, and enhanced cell attachment complementing CNT, producing a silk sericin-CNT hybrid ink (SSCNT). SSCNT is synthesized by dispersing CNT nanoparticles to sericin solution extracts (Figure 3a), and the ink may be fabricated into electrodes or circuit patterns via printing and textile dyeing. Wearable electrode application can be achieved through conductive textiles fabricated by dyeing commercial textiles in SSCNT ink. Free-standing SSCNT films were fabricated by drop-casting to examine SSCNT dispersion. Electrical conductivity was 42.1 ± 1.8 S cm^−^^1^, which is comparable with CNT inks from alternative dispersants such as sodium dodecyl benzenesulfonate, silk fibroin, and bovine serum albumin. Cell incubation confirmed biocompatibility, which proved the lower cell toxicity of SSCNT compared to stand-alone CNT. Textile electrodes fabricated from SSCNT-dyed conductive fabric successfully employed as ECG sensors, demonstrating high flexibility and conductivity of SSCNT as highly stable and biocompatible conductive inks.

Kwon et al. introduced a new form of hybrid electronics utilizing functionalized conductive graphene (FCG) and Ag printed dry electrodes [56]. The all-printed, nanomembrane hybrid electronics (p-NHE) utilize FCG as a protective oxidation barrier to the underlying Ag and function as sensing electrodes. The layered aerosol-jet-based printing (AJP) method was applied to achieve direct ink deposition for varying viscosity without the additional use of screens or patterning masks (Figure 3b) at ultra-low thickness (20 nm). The structural reliability and bending property of the FCG electrode were proven sufficient, stretching up to 60%, which exceeds maximum skin stretchability (30%) during daily activity. The serpentine patterned mesh structure of the FCG electrode also demonstrated high skin formality, mitigating signal disruption due to motion artifacts. The p-NHE demonstrated usability in a wide range of human-machine interfaces by collecting high-quality EMG mapping data with low SNR (9.5 dB). Li et al. proposed a conductive CNT-TPU composite thin film patterned textile substrate for dry textile electrodes. The CNT-TPU thin films are fabricated by TPU solution infiltration onto layered CNT sheets, which are then cut into electrode shapes by laser cutting (Figure 3c) [57]. The conductive thin film was then transferred onto textile substrates in a heat press machine. For electromechanical property evaluation, samples ranging between 5–10 μm in thickness were taken. Strain measurements ranged from 20–30%, which is enough to accommodate fabric strain experienced during normal human activity (~20%). Impedance measurements ranged from 3.4 × 10^4^–1.4 × 10^7^ Ω at 200 Hz, aligning with values observed in comparable textile-based reference electrodes. The skin–electrode impedance values demonstrated are low enough to transduce ECG signals with SNR comparable to golden-standard Ag/AgCl gel electrodes. ECG signals collected by a CNT-TPU electrode lined sleeve demonstrated good signal quality while the wearer performed arm flexion.

Sun et al. propose a simple, general approach to fabricating multifunctional wearable electrodes by implementing laser-induced porous graphene (LIG) with super-templated silicon elastomer sponge substrates [58]. Direct CO_2_ laser patterning on PI substrate allows exposed PI surfaces to convert directly to porous graphene by a photothermal process, which is simpler and cost-effective than conventional microelectronic fabrication methods such as lithography, vacuum deposition, and surface etching (Figure 3d). The porous microstructure of both LIG and silicone substrate allows for great water-vapor permeability, significantly suppressing the risk of skin inflammation for the wearer. The resulting LIG electrode measures ~20 μm in thickness, withstand up to 330% deformation before breakage and retains original electrical properties when released from 60% deformation. Electrical properties such as resistivity and contact impedance proved comparable to gold-standard gel electrodes (resistivity: 10.96 Ω/sq, contact impedance: ~17 kΩ at 100 Hz). To prove the viability of LIG on silicon electrode applicability to on-skin dry electronics, in vivo testing of ECG, EMG, and EEG was performed. All signals demonstrated high stability with ECG SNR of 24.1 dB, comparable to ECG signals from Ag/AgCl gel electrodes. Carbon black (CB) is an old and cost-effective carbon nanomaterial that has recently been re-discovered as potential biosensing material. CB exhibits excellent electrical properties and offers additional benefits such as solvent dispersibility, facile fabrication, and high surface area [52]. Cheng et al. proposed PDMS-CB carbon-based electrodes for continuous, long-term, and stable ECG monitoring [59]. The PDMS-CB composite is fabricated by mold casting, which is then transferred by tape onto a surface. The fabricated thin film can be laminated onto surfaces with complex curvature, increasing conformability and reducing motion artifacts. Conductivity was measured to vary depending on varied conductive nanoparticle filler content, ranging from 1 × 10^−10^ to 1 × 10^−3^ S/m, reaching saturation beyond 10% filler content. ECG was measured from the left chest to evaluate signal quality compared to Ag/AgCl gel electrodes. Contact impedance ranged between 13–18 kΩ at 10–10,000 Hz from ECG data. The “PQRST” characteristic analysis of ECG signals proved the similarity of the PDMS-CB electrode data to conventional rigid electrodes, indicating the ability to measure ECG for biological monitoring continuously.

### 2.4. Conductive Polymer-Based Electrodes

Since their discovery in the 1970s, conductive polymers (CP) have attracted interest in biosensing applications due to unique properties such as lightweight, flexibility, scalability, corrosion resistivity, and ease of customization with various co-additives to suit particular needs [64,65]. Polymer nanocomposites (PNC) refer to CP materials combined with other nanomaterial types to improve electrochemical significance. Such highly electroactive compounds are developed with the special properties of individual compounds having a synergistic effect. Among various CP types, poly(3,4-ethylenedioxythiophene) polystyrene sulfonate (PEDOT:PSS) is considered one of the most successful and widely studied materials for biological applications, which commonly utilize an aqueous dispersed formulation [66]. PEDOT:PSS demonstrates a combination of electronic and ionic conductivity, commercial availability, flexibility, and biocompatibility [67]. Another CP that has gained popularity as an epidermal biosensing electrode material is polypyrrole (PPy). PPy is an electrode deposited polymer that can be doped with various additives for property altercations resembling PEDOT:PSS [68,69]. This section will investigate notable CP compounds in dry electrode applications as well as various electromechanical properties optimized using various co-additives and fabrication methodologies. Table 3 summarizes conductive polymer-based electrodes.

**Figure 4:**
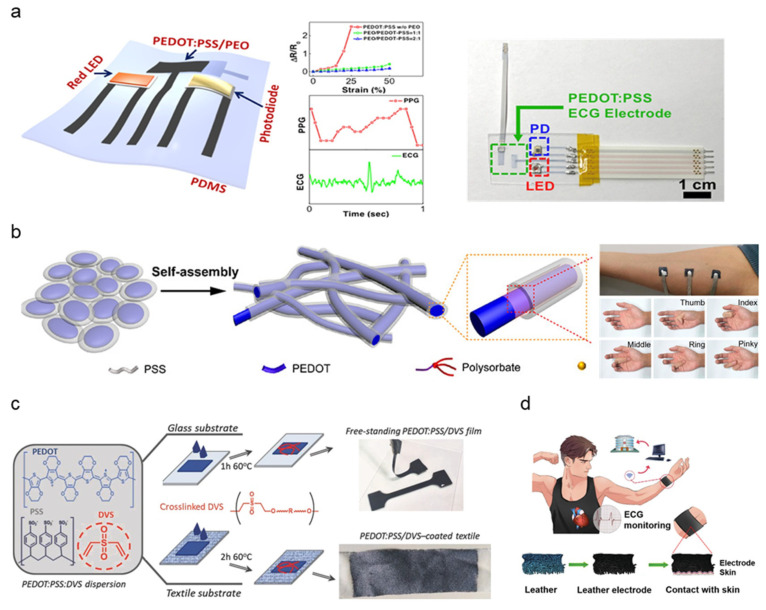
Schematic diagram of polymer-based electrode synthesis and clinical demonstrations; (**a**) Assembled form-factor diagram and photograph of PEDOT:PSS/PEO electrode alongside strain/resistance measurement, ECG and PPG signal data (Reprinted with permission from *ACS Appl. Mater. Interfaces.* 2021, *13*, 18, 21693–21702, Copyright 2021, ACS) [70]. (**b**) Diagram of fibrous PEDOT:PSS treated with glycerol and polysorbate layering implemented in a demonstrative photograph of EMG data collection (Reprinted with permission from *ACS Mater. Lett.* 2021, *3*, 9, 1385–1393, Copyright 2021, ACS) [71]. (**c**) PEDOT:PSS/DVS fabrication schematic into coated textile and free-standing film electrodes (Reprinted with permission from *Adv. Mater. Technol.* 2018, *3*, 1700322, Copyright 2021, Wiley) [72]. (**d**) Applications of PPy coating leather electrode to remote healthcare and cardiovascular disease monitoring (Reprinted with permission from *Adv. Electron. Mater.* 2020, *6*, 2000259, Copyright 2020, Wiley) [76].

While the most common form of conductive polymer for bioelectric sensing is PEDOT:PSS, mechanical properties can be optimized by blending additional polymer additives. The majority of PEDOT:PSS-based polymer dry electrodes adopted a facilitating matrix to combat the mechanical rigidity of PEDOT:PSS and fabrication into a stretchable thin-film structure to be used as dry electrodes with innate adhesive properties. Lo et al. synthesized PEDOT:PSS thin films blended with polyethylene oxide (PEO) patterned using inkjet printing [70]. (Figure 4a). The addition of PEO and ethylene glycol (EG) solvent removes the brittleness and yields improved stretchability. PEDOT:PSS/PEO film patterns were inkjet-printed, demonstrating low sheet resistance (84 Ω/sq) and high stretchability (<50%). ECG and PPG signals obtained by PEDOT:PSS/PEO electrodes exhibited distinguishable peaks, measuring ventricular activity such as heart rate. A delamination-resistant imperceptible bioelectrode (DrIE) developed by Tang et al. introduces a low-cost solution processing of PEDOT:PSS, glycerol, and polysorbate polymers for a perturbation-resilient, breathable, and delamination-resistant dry electrode [71]. Glycerol blending reduced electrostatic interactions between PEDOT and PSS, allowing PEDOT to partially aggregate, forming a conductive network while maintaining PSS dispersion to generate an ultra-thin matrix (thickness: 20 μm) (Figure 4b). Additional polysorbate doping achieves high conductivity under large strain by bridging the PEDOT and PSS domains (conductivity range: 70~140 S/cm, strain endurance: 90%). The DrIE film, fabricated via drop-casting onto a PDMS supporting substrate, notably showed lower skin impedance (7 × 10^2^–10^5^ Ω/cm^2^ at 10–10^5^ Hz) on wet surfaces compared to dry surfaces. Such moisture-resistant properties are induced by an increased skin–electrode area with potential application to long-term monitoring under electrode exposure to fluids such as secreted sweat or environmental humidity.

A self-adhesive conductive polymer (SACP) developed by Tan et al. incorporates a mixture of PEDOT:PSS crosslinked with supramolecular solvent (SMS) and an elastic polymer network [74]. SMS additives such as citric acid and cyclodextrin improved mechanical properties by increasing fracture strain and lowering Young’s modulus. The elastic polymer network is synthesized by crosslinking polyvinyl alcohol (PVA) with glutaraldehyde (GA) to improve stretchability and elasticity. The resulting SACP exhibits strong adhesion to substrates due to the synergy of weak surface interactions, demonstrating scalable application to flexible wearables. SMS, PVA, and GA doping to PEDOT:PSS achieves highly controllable electroconductivity between 1–37 S/cm. Maximum conductivity was achieved by a 36.3% PEDOT:PSS mass ratio. Synthesized SACP ink was molded into thin-films by several fabrication methods such as microfluid molding, drop casting, and spin coating. The resulting SACP thin-film was assessed for EMG signal collection, demonstrating synchronous deformation with the skin and signal quality comparable to commercial Ag/AgCl electrodes. Repetitive testing also demonstrated no apparent signal degradation upon cycling the SACP electrodes ten times under EMG collection setups. Del Agua et al. proposed divinyl sulfone (DVS) as an alternative PEDOT:PSS crosslinking agent, developing a PEDOT:PSS/DVS formulation easily processed into free-standing films along with textile electrodes [72]. The addition of DVS increased break value strain with a lowered Young’s modulus (break strain: <15 ± 0.4%, Young’s Modulus: 19.3 ± 0.7 MPa), counteracting the brittleness of PEDOT:PSS. Free-standing PEDOT:PSS/DVS films were fabricated by drop-casted agents being peeled off a glass substrate (Figure 4c). Textile electrodes were fabricated to improve wearability and enforce mechanical robustness by depositing PEDOT:PSS/DVS to a textile substrate rather than glass. The PEDOT:PSS/DVS-coated textile demonstrated stable electrical properties upon cyclic testing (100 times at 20% strain). Both free-standing and textile electrodes demonstrated improved contact impedance (1.5 × 10^2^–1 × 10^5^ Ω at 0.1–10^5^ Hz) in comparison to commercial gel electrodes. ECG data obtained from PEDOT:PSS/DVS textile electrodes demonstrate the development of robust wearable electronics in smart clothing for daily health monitoring.

Zhang et al. proposed a unique electrode form-factor utilizing polypyrrole (PPy) polymerized onto a goat-leather substrate [76]. Animal-derived leather consists of a micro collagen fiber cluster that highly matches human skin structure, improving conformal contact while avoiding complex surface construction of synthesized micro-nano structures. Leather also features good breathability and antibacterial properties, making it highly biocompatible. PPy is directly deposited on leather via in situ polymerization with the addition of p-Toluene sulfonic acid (PTSA) to improve electrical conductivity and strengthen the adhesion between PPy and leather fibers. Impedance measurements at a frequency matching the average human heart rate (1 Hz) proved PPy to have a lower impedance (28 Ω cm^−2^) than commercial electrodes (64 Ω cm^−2^). Upon washing and drying, the resistance of PPy electrodes increased by a marginal amount (18.5–24.7 Ω cm^−2^ at 1 Hz), significantly lower than commercial electrodes (63.3 Ω cm^−2^ at 1 Hz). ECG signal collection was performed using a wrist strap lined with PPy-leather electrodes, successfully collecting signals with no significant deviations from commercial electrodes (Figure 4d). Like SSCNT ink from the Liang group, Meng et al. developed a dry polymer electrode based on a silk-fibroin (SF) layer for enhanced adhesion. A PPy conductive layer was combined with layers of acid-modified silk (AM-SF)/cellulose nanocrystal (CNC) film and a Ca-modified SF adhesive layer [75]. A diazonium coupling reaction stimulated the positively charged PPy, which promoted absorption and intercalation within the negatively charged silk network to form the PPy@AM-SF/CNC electrode film. To achieve film stretchability resembling human skin, glycerol was incorporated into the PPy@AM-SF/CNC films for improved stretchability (maximum elongation surpassing 100%). The PPy@AM-SF/CNC also outperformed commercial Ag/AgCl electrodes in contact impedance at varying thickness ranges (21–80 μm). Contact impedance for PPy@AM-SF/CNC exceeded Ag/AgCl electrodes (1 × 10^2^–4 × 10^6^ Ω at 0.1–1 × 10^5^ Hz). Finally, biopotential detection was demonstrated by ECG and EMG which were highly comparable to Ag/AgCl signal quality along with remarkable stability upon skin deformation such as twisting, compression, and stretching. EMG signals were able to distinguish various hand gripping forces and opening/closing motion through peak-to-peak amplitude observations.

### 2.5. Other/Hybrid Electrodes

**Figure 5:**
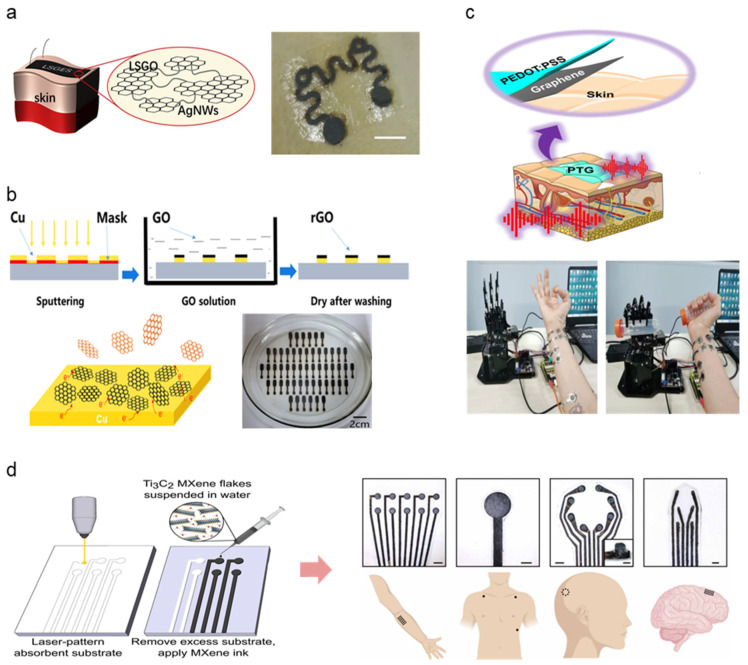
Hybrid material selections for high-performing novel dry electrode architecture; (**a**) Schematic image of LSGO layer linkage via AgNW connectors and adhesion on the hairy skin surface (Reprinted with permission from *Carbon* 2020, *156*, 253–260, Copyright 2020, Elsevier) [77]. (**b**) rGO electrode fabrication demonstrated via GO reduction mechanism, photograph of assembled electrodes under scalable fabrication settings (Reprinted with permission from *Carbon* 2020, *164*, 164–170, Copyright 2020, Elsevier) [79]. (**c**) Schematic illustration and demonstration photograph of PEDOT:PSS and graphene layered electrode for EMG detection (Reprinted with permission from *Nat. Commun.* 2021, *12*, 4880 (2021), Copyright 2021, Nature Communications) [82]. (**d**) MXene electrode fabricated via laser patterning and photograph of assembled 3D EEG sensor array demonstrated on the human subject (Reprinted with permission from *Sci. Transl. Med.* 2021, *13*, 612 (2021), Copyright 2021, Science Translational Medicine) [81].

A range of material selection for the fabrication of high-performance soft, dry electrodes has been observed, categorized into metal, carbon, and polymer divisions. This subsection will observe recent dry electrode studies implementing a combination of such material selections to improve dry electrode performance. Table 4 summarizes other and hybrid electrodes. Metal and carbon substrates, as outlined above, are widely applied as electrode base material in the form of nanoparticles dispersed in flexible, conformable elastomer substrates such as PDMS and polyurethane (PU). Qiao et al. combined laser-scribed graphene oxide (LSGO) and Ag-nanowires (AgNW) to form graphene oxide electronic skin (GES) [77] (Figure 5a). The AgNW connects flaky LSGO particles, demonstrating excellent signal detecting performance for ECG and EEG (resistivity: 700 Ω, impedance: 20–100 kΩ at 10–10,00 Hz). Lee et al. used AgNW and CNT combination embedded into PDMS to develop a portable, earphone-type biosensor for electrophysiological detection via EEG signal collection [78]. The addition of AgNW demonstrated improved elastomeric composite conductive when mixed in appropriate amounts (5 wt% of CNT). Li et al. demonstrate the scalable manufacturability of metal-carbon electrodes by fabricating reduced graphene oxide (rGO) electrodes coated with a patterned Cu layer via magnetron sputtering at room temperature [79] (Figure 5b). Active metals such as Cu lower the surface redox potential of rGO without compromising skin–electrode impedance. The mass-produced arrays were set up as multi-channel electrode arrays for EMG, EOG, and EEG measurements, demonstrating long-term electro potential signal monitoring.

**Table 4:** Summary of other/hybrid soft–dry electrodes.

Electrode Material	Manufacturing Method	Conductivity/ Resistivity	SNR/Contact Impedance	Stretchability	Thickness	Ref.
AgNW, Laser scribed graphene oxide (LSGO)	Mixture volatilization on transfer paper to form a thin film	700 Ω	20–100 kΩ (@ 10–1000 Hz)	1–4%	N/A	[77]
Cu/rGO	Magnetron sputtering	N/A	100 k–900 kΩ (@ 10–1000 Hz)	N/A	0.4–4.9 μm	[79]
Graphene, PEDOT:PSS	Spin-coating	~24 Ω/sq (4142 S/cm)	4 × 10^2^–7 × 10^4^ Ω (@ 10–10^5^ Hz)	40%	~100 nm	[80]
MXene	Spray, Spin, Dip Coating, Direct writing, IJP	15,000–20,000 S/cm	241.4 ± 14.7–1343.3 ± 81.6 Ω (0.05–3 mm)	N/A	N/A	[81]
Nanofiber membrane (PVDF/PEDOT:PSS), CB/rGO	Electrospinning	2.5 × 10 Ω/sq	2 × 10^2^–7 × 10^5^ Ω	10–50%	100 μm	[82]
CNTs/AgNw/PDMS	Sonication	N/A	10^5^ Z (@ 10 Hz)	30%	200 μm	[78]
PEDOT:PSS covering laser-induced graphene (LIG)	Spray coating, Laser cutting, mask peel-off	13.1–33.5 Ω/sq	17.4 U/sq 385 kU (@ 10 Hz)	N/A	N/A	[83]

PEDOT:PSS has been noted as a widely popular base material in the polymer subsection due to innate conductive properties and compatibility with additive materials. Here, we demonstrate several carbon-polymer compound electrodes taking full advantage of such features. Zhao et al. introduced dry electrodes exhibiting good skin adhesion, low sheet resistance, high transparency, and electromechanical stability attributed to the synergistic effect between graphene and PEDOT:PSS [80]. (Figure 5c) The strong π-π interaction between the two materials induces a high-degree molecular ordering of PEDOT and graphene charge transfer. Zahed et al. proposed a similar graphene-PEDOT:PSS design by spray-coating PEDOT:PSS onto a laser-patterned LIG surface (PP/LIG) for improved conductivity [83]. PEDOT:PSS acts as linkers between the porous LIG surface and interconnects LIG flakes, mitigating crack formation during cyclic bending. The robustness of the fabricated PP/LIG was demonstrated via repeated rubbing after submerging the PP/LIG in water for several days to demonstrate adhesivity under aqueous conditions, demonstrating the electrode’s ability to be used in long-term signal monitoring. Huang et al. combined PEDOT:PSS with polyvinylidene difluoride (PVDF) to form a nanofiber layer via electrospinning, combined with a carbon electrode layer synthesized from nano dispersed carbon black (CB) and rGO [82]. PEDOT:PSS prevented bead formation within the fiber microstructure, enhancing mechanical integrity as well as electrical conductivity. The resulting nanofiber carbon electrode exhibits high stability and improved electrical properties. First introduced in 2011, MXene is a two-dimensional material compound consisting of transition metal carbides, nitrides, or carbonitrides. MXene’s biological application has been highlighted in recent studies due to its innate metallic conductivity combined with hydrophilicity [84]. Driscoll et al. developed MXtrodes, a class of soft, high-resolution, large-scale bioelectronic interfaces by scalable solution processing of Ti3C2 MXene [81]. The hydrophilic properties of MXene enable processing using simple water-based ink, offering a wide range of fabrication methodologies (e.g., spray, spin, dip coating, direct writing, IJP). The water-based MXene ink was doped onto a laser patterned cellulose-polyester porous substrate, allowing for rapid prototyping and geometric customizability (Figure 5d). The skin–electrode impedance of MXtrodes was evaluated, demonstrating superior electroconductive properties compared to commercial Ag/AgCl electrodes (6.6 ± 2.9, 4.9 ± 2.6 kΩ at 1 kHz). Epidermal sensing was justified by EMG, ECG, and EOG signal acquisition. The study further proposes using MXtrodes as implantable sensing and stimulation applications such as intraoperative electrocorticography (ECoG) and EEG, along with clinical imaging assessed via MRI compatibility. The extensive applicability of such novel materials opens extensive pathways for exploring alternate bio-applicable materials and harnessing distinct material properties to achieve flexibility, conductivity, and biocompatibility suitable for high-performance wearable physiological monitoring devices.

## 3. Physical Adhesion

### 3.1. Significance of Adhesion in Soft–Dry Electrode Performance

Proper adhesion is one of the most important factors for successfully measuring physiological signals by reducing motion artifact and lowering skin–electrode contact impedance, which are key factors for high-quality signal acquisition [85,86,87]. Motion artifact is a noise in the signal that is due to the movement of the electrode concerning the human skin caused by the motion of the subject [86], which affects the signal quality of the electrode. Skin–electrode contact impedance is a response of a skin region to the externally applied sinusoidal current [88]. The skin–electrode impedance largely increases when an air gap exists between the skin–electrode interfaces as the air gap forms a dielectric layer [87]. Because nearly all biopotentials such as EEG, ECG, and EMG have low-frequency range and extremely low amplitude and are measured at mV level with on-skin electrodes [89], the signal acquisition could be strongly disrupted by noises caused by motion artifacts and the impedance change, which emphasizes the importance of adhesion in biopotential measurement. To obtain a better quality of the signal by reducing motion artifacts and lowering the skin impedance, the type of adhesives and the conformal contacts must be considered because proper adhesion reduces a motion artifact [90,91], and the conformal contact not only reduces motion artifact but also decrease the contact impedance [91]. Moreover, soft–dry electrodes with proper adhesion can benefit long-term usage due to the reduction of motion artifact. In this chapter, adhesion will be categorized into two categories: (a) adhesive and (b) conformal contact, in which the adhesive section will focus on the adhesion mechanism to reduce the motion artifact, and the conformal contact section will focus on the adhesion mechanism to reduce the contact impedance.

### 3.2. Adhesive

Soft–dry electrodes used adhesives that could be categorized into two methods depending on how they interact with skin. Dry adhesives are the type of adhesive that uses micro-structured electrodes to form a dry adhesive. Pressure-sensitive adhesives use or are made with a self-sticking adhesive whose strength increases upon allied pressure. Table 5 summarizes great examples of dry adhesives and pressure-sensitive adhesives for the soft–dry electrode.

#### 3.2.1. Dry Adhesive

**Figure 6:**
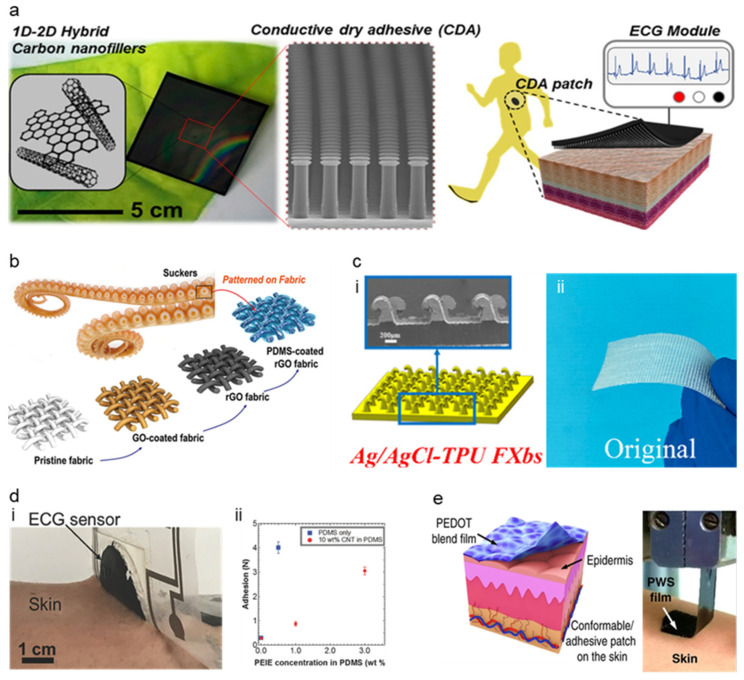
Types of various adhesives for dry electrodes. (**a**) Image of Gecko-inspired structure. (Reprinted with permission from *ACS Nano* 2016, *10*, 4770–4778, Copyright 2016 ACS) [92]. (**b**) Octopus-inspired Graphene Fabric Sensor. (Reprinted with permission from *ACS Appl. Mater. Interfaces* 2019, *11* (18), 16951–16957, Copyright 2019 ACS) [105]. (**c**) Fructus Xanthii-inspired dry bioelectrode. (Reprinted with permission from *ACS Appl. Mater. Interfaces* 2021, *14*, 4, 6028–6038, Copyright 2022 ACS) [95]. (**d**) Gel-less sticky ECG patch (Reprinted with permission from *Adv. Healthc. Mater.* 2017, *6*, 1700495, Copyright 2017 Wiley) [96]. (**e**) Ultra-Thin PWS blend film. (Reprinted with permission from *Nat. Commun.* 2020, *11*, 4683, Copyright 2020 Nature Communications) [73].

The first type of adhesive is called dry adhesives, referred to as a non-sticking electrode gaining adhesion due to structure modification mostly on a micro-nano scale. The recent development of dry adhesives for soft electrodes focuses on the nature-inspired structure. Gecko-inspired micropillars are one widely used nature-inspired structure for dry adhesion [92,93,98,99,100,101,102,103]. The foot of the gecko has millions of hydrophobic seta, and these setas achieve strong adhesion through van der Waals force. It is researched that hydrophobicity of the micropillars and increase of adhesion forces due to an increase of surface density shows that the micropillars achieve adhesion with van der Waals force which more specific research about Gecko adhesion mechanisms are explained in this literature [101]. Kim et al. [92] introduced a gecko-inspired 1D-2D hybrid carbon nanocomposite-based dry adhesive for ECG electrodes shown in Figure 6a. This ECG electrode is fabricated by deep etching a Si mold and fabricating a dry adhesive by casting 1D-2D hybrid carbon nanofillers on the Si mold. This nanofiller was composed of higher dimensional carbon material (carbon black, nano graphite, and graphene nanopowder) as an aid filer to a 1D carbon nanotube, showing the structural modification of substrate could replace the glue while maintaining its adhesion quality. This gecko-inspired micropillar structure has low volume resistance and high normal adhesion force of ~1.3 N/cm^2^, even on rough human skin, proving its versatile application. The electrode repeated the attach-detach cycle (the surface being cleaned after six cycles) 30 times without losing its adhesion showing semipermanent reusability. The performance of the electrode was not significantly hindered by a 30% stretch of the surface, showing ~14% decreased conductivity.

High adhesion of gecko-inspired micropillar adhesives showed electrode’s motion artifact resistance and efficient reusability, proving high performance as the soft–dry electrode. Dirk-M et al. [93] have proposed microfibrillar adhesive film that uses microfibers instead of micropillars. This microfibrillar structure was fabricated with poly (dimethylsiloxane) (PDMS) microfibers, and mushroom-shaped vinylsiloxane (VS) was added to the tip so the tip could make crosslink on the skin and significantly increase the skin adhesion. The microfibrillar film showed strong adhesion of 18 kPa and achieved a 59.7 SNR noise ratio due to the signal amplification of the microfibrillar structured film. This structure has shown great usage upon a slightly rough and soft surface due to its mushroom-shaped viscous tip. Moreover, there were trials to overcome the gecko-inspired adhesive’s weak point that the gecko-inspired adhesive losses the van der Waals force when the surface is wet [94], especially in an environment like underwater. As a solution, there was an attempt to fabricate dry adhesive with octopus-inspired microsucker that uses vacuum state formation inside the suction cup due to its structural collapse showing strong, reversible adhesion even on the wet and rough surface [94,104,105,106]. Chun et al. [105] show the possibility of dry adhesion from the miniaturized octopus-like microsucker design. The octopus-like patterned graphene-coated fabric allows it to adhere to both wet and dry skin properly. The proper adhesion enabled the device to sense the ECG and wrist-bending motions even in wet conditions. (Figure 6b) Chun et al. [94] also researched octopus pattered (OP) carbon-based conductive polymer composite. This OP film was fabricated by silicon mold with microholes. Its dome-like protuberance structure of octopus sucker made this adhesive adhere to skin strongly without needing external adhesive such as glue. The adhesion test reported that the OP film has 5.24 N cm^−2^ of adhesion strength on silicon and 1.89 N cm^−2^ of adhesion strength on the skin replica and did not leave any residue on the skin even with effortless detachment. The comparison of ECG signal and bending motion of wrist measurement under dry and wet conditions showed no significant difference in the signal. Therefore, the strong adhesion of the octopus patterned film showed high performance as a soft–dry electrode not only in dry conditions but also in wet conditions. Other than gecko/octopus inspired structure, the hybrid structure of hexagonal tree frog toe pad-inspired microchannels with octopus [104], Fructus xanthii-inspired that is resistant to the motion artifact due to its high dynamics friction [95], (Figure 6c) Dytiscus lapponicus inspired barb structure [107], etc. could provide a good example of a dry electrode with the structurally modified dry adhesive applied on it.

#### 3.2.2. Pressure-Sensitive Adhesive

The second type of adhesive is a pressure-sensitive adhesive (PSA) which forms a bond with the skin due to applied pressure. Polymeric materials are one kind of PSA that uses viscoelastic energy dissipation of the material to create adhesion to the skin [73,96]. There was an attempt to fabricate PSA simply but effectively by mixing ethoxylated polyethyleneimine (PEIE) with polydimethylsiloxane (PDMS) and enhancing its conductivity by mixing multiwall carbon nanotubes (MWCNT). Yamamoto et al. [96] proposed a gel-less sticky adhesive with a high adhesive conductive polymer. To get the high adhesive conductive polymer, PDMS was mixed PEIE for enhanced adhesion, and PDMS was also mixed with MWCNT giving conductivity to the layer with a ratio of 10 wt% MWCNT and 3 wt% PEIE in PDMS, cured at 100 degrees Celsius. Given adhesion and conductivity to PDMS for efficient adhesion and higher signal quality, the PDMS-PEIE-MWCNT film electrode demonstrated its high performance as the soft–dry electrode. PDMS-PEIE-MWCNT film electrode showed adhesion strength of 3 N cm^−2^ to the skin, and this gel-less sticky adhesive was stable and able to use after 90 days of exposure in air and did not lose its adhesion up to 100 cycles of ECG signal detecting, which proved efficient adhesion and long-term stability of the electrode.

There also was an attempt to directly grant adhesiveness to the common form of conductive polymer, PEDOT:PSS, by solution processing. Zhang et al. [73] proposed a high-quality signal acquisition self-adhesive long-term motion monitoring PWS blend film. PEDOT:PSS, waterborne polyurethane (WPU), and D-sorbitol were solution-processed and molded into a thin film for its efficient adhesion. (Figure 6e) The characteristic of the PWS film shown by the SEM image shows grainy nanoscale morphology with a grain size of −100 nm. The Young’s modulus and stress could be modified due to the wt% of PEDOT:PSS loading where low wt% of PEDOT:PSS showed the lowest Young’s modulus and lowest stress when the strain was applied. At the loading of 38 wt% D-sorbitol, the PWS films showed max adhesion strength of 0.41 and 1.44 N/cm on skin and glass. With PWS blend film, ECG, EMG, and EEG were measured, showing outstanding signal quality due to efficient adhesion. The ECG signal collected with PWS electrodes had RMS noised around 25 μV, which is a lower value compared to that of well-known Ag/AgCl gel electrodes (28 μV) and the noise increasing rate of PWS electrodes (27 μV after 1 week) was also lower than that of Ag/AgCl gel electrode (32 μV after 1 week) showing long-term measuring stability. Another kind of PSA also adopted a nature-inspired mechanism such as mussel-inspired self-adhesive [97], showing strength in long-term measurement along with resistance to the motion artifact. The use of dopamine, inspired by how mussels adhere to the surfaces, improved the adhesion strength as PSA and high resistance to sweaty conditions. Xu et al. [97] demonstrated a mussel-based elastomer that is highly adhesive and self-healing for the bio-interfacial electrode. For efficient adhesion, dopamine was applied to elastomers as pendent groups. Mussels use adhesive proteins to attach to various surfaces by a solidified protein forming adhesive plaques that are strongly adhesive and durable. 3,4-dihydroxyphenylalanine (Dopa) is the key material for mussel-inspired adhesion mechanism forming H-bond [108], π-π electron interaction [109], cation-π interaction [110], and etc. Reliable adhesion with tissue comes from dihydroxy [108], forming a strong hydrogen bond with protein. For the highly adhesive elastomer, dopamine (DMPA-DA) was mixed into a urethane-based polymer resulting in adhesive strength of ~62 kPa and ~54 kPa of strength after being soaked in water 24 h proving. The signal quality was similar to EMG measurement of commercial Ag/AgCl gel electrodes in dry conditions. While the commercial electrode was detached after 10 m of running, the mussel-inspired highly adhesive electrode did not lose either adhesion or signal even for 30 m of running in sweaty conditions, showing high performance as the soft–dry electrode.

### 3.3. Conformal Contact

**Figure 7:**
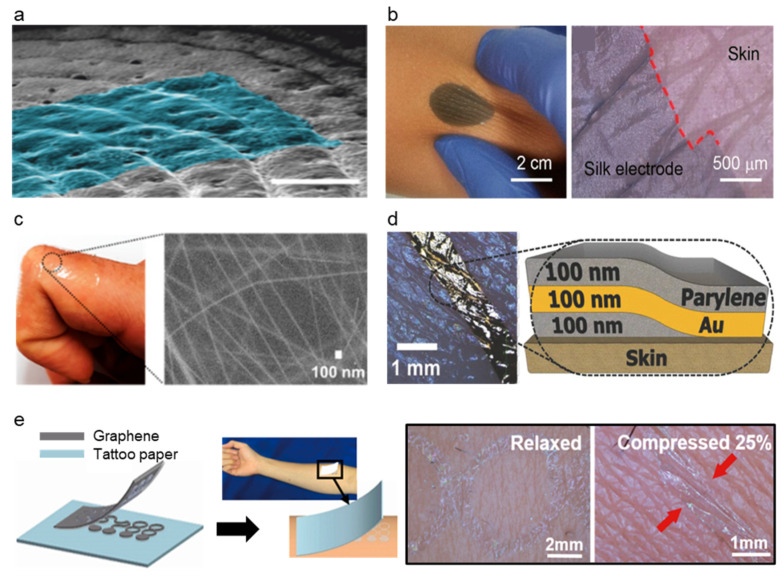
Approaches to promote conformal contact of the electrode. (**a**) Ultra-thin temporary tattoo electrode with ultra-conformability. (Reprinted with permission from *Adv. Sci.* 2018, *5*, 1700771, Copyright 2018, Wiley). (**b**) Conformal Silk-Based Electrode. (Reprinted with permission from *ACS Nano* 2021, *15*, 6, 9955–9966, Copyright 2021, ACS). (**c**) AgNW embedded a-PDMS matrix. (Reprinted with permission from *Nano Letters* 2018, *18* (7), 4531–4540, Copyright 2018, ACS). (**d**) Ultra conformable sub 300 nm dry thin film. (Reprinted with permission from *Adv. Funct. Mater.* 2018, *28*, 1803279, Copyright 2018, Wiley). (**e**) Simple skin–electrode mechanosensing structure (SEMS). (Reprinted with permission from *ACS Nano* 2017, *11*, 8, 7634–7641, Copyright 2017, ACS).

As abovementioned, conformal contact is one of the essential factors as soft electrodes with conformal contact on the skin reduce the impedance and improve the quality of the signal that we gain from the electrode. Conformal contact of electrode-skin enables electrodes to maximize the contact area of the electrode to the skin. It is well shown in Figure 7a where the shape of the electrode conforms to the shape of the skin and changes its shape accordingly as the skin deforms and changes its surface configuration. The increased contact area increases the capacitance that improves the signal quality [87]. Moreover, conformal contact reduces the electrode-skin impedance, which could greatly improve electrode performance shown with a signal-to-noise ratio (SNR) [87]. Conformal contact can also improve the signal quality by reducing the motion artifact by adopting similar bending stiffness of the electrode to that of the stratum corneum (the uppermost layer of the skin where the sensor directly has contact) so that the bending of the electrode could follow the movement of skin [111]. Recent studies on conformal contact for soft–dry electrodes are summarized in Table 6.

#### 3.3.1. Material Modification

Modifying material characteristics such as Young’s modulus, bending stiffness, etc., is one of the methods to improve the conformal constant of skin–electrode [111]. Electrode-skin impedance could be lowered when the modulus of the electrode is similar to the skin [87]. Modification of conductive polymer, PEDOT:PSS, was succeeded by enhancing its conductivity and lowering Young’s modulus by blending it with the glycerol [112]. Glycerol lowers Young’s modulus by disrupting the hydrogen bond in the amorphous phase and weakening the interaction between chains. Li et al. [112] proposed a conformal silk-based electrode with sweat tolerance shown in Figure 7b by coating glycerol mixed PEDOT:PSS solution to the glycerol-plasticized silk fiber mat. The optimized amount of glycerol on the PEDOT:PSS solution was 15% (labeled as Silk-P-15G for the electrode with optimized PEDOT:PSS-glycerol solution). Silk-P-15G decreased Young’s modulus from ~220 to <3 MPa and increased its stretchability from <10% to ~340% as the glycerol was added. The conductivity was also increased from ~0.18 S/cm to ~24 S/cm. The addition of the glycerol and annealing also enhanced the water and sweat stability of the electrode. Silk-P-15G showed excellent electrode-skin impedance comparable to that of commercial ionic gel electrodes. The high stretchability of the electrode allowed it to record stable signals under large deformation conditions. The electromyography (EMG) signals recorded under different deformation lengths showed comparable SNR and amplitudes to the commercial gel electrode. Tang et al. [71] reported delamination-resistant imperceptible bioelectrode (DrIE) for electrophysiological signals measuring devices. DrIE was made of solution processing of PEDOT:PSS, glycerol, and polysorbate. The base of the film was PEDOT:PSS as an intrinsically conductive polymer, and the glycerol reduced electrostatic interactions between PEDOT and PSS, adopting proper mechanical flexibility [112], and polysorbate allowed the glycerol-doped PEDOT:PSS to have high fracture strain. The DrIE had an adhesion force of ~1.2 N, Young’s modulus of 0.8 MPa, and stretchability of 80%. Due to its high conformity, DrIE showed 35.23 ± 1.94 dB of SNR, comparable to that of commercial Ag/AgCl electrode (34.72 ± 0.68 dB), proving its high performance as the soft–dry electrode. Another attempt to directly modify PDMS was made to increase its conformal contact of the AgNW-PDMS electrode. Kim et al. [113] proposed silver nanowire (AgNW), triton X embedded PDMS matrix (a-PDMS matrix) electrode as strain and ECG sensor (Figure 7c). The PA-PDMS matrix showed significant improvement in signal sensing performance compared to the PDMS-based sensor due to its conformability and stretchability on the electrode-skin relationship. Triton X, a nonionic surfactant, was used for the physical property modification of PDMS such as young’s modulus, adhesiveness, and viscoelasticity. Triton X could make PDMS have heterogeneous cross-linked networks of polymer chains by inhibiting its cross-linking reactions. A4-PDMS_40, which contained 0.4 wt% Triton X showed a young’s modulus of 40 ± 5 kPa and failure strain of >400%, while the untreated PDMS_40 showed 480 ± 30 kPa and failure strain of 230%. Low Young’s modulus and viscoelasticity of a-PDMS matrix showed high performance as an electrode showing ten times lower impedance than the untreated PDMS sensor. The signal of a4-PDMS_40 shows lower noise than that of the commercial gel-based ECG sensor.

#### 3.3.2. Structural Modification

While the material modification of the electrode could achieve conformal contact, structural modification of the electrode could also achieve conformal contact as they have contact with skin [80,111,116]. The ultrathin film is the most commonly used method for structural change of the electrode (Figure 7d). The ultrathin film adheres to the substrate by van der Waals force [117], and therefore, the thin-film electrode makes conformal contact with the elastomer and the skin. Simply by sandwiching a thin Au layer between thin layers of parylene, conformal contact of the electrode was achieved by Someya et al. [111]. Someya et al. introduced an ultra-conformable, sub-300 nm dry, thin-film electrode shown in Figure 7d. The bending stress of this electrode is around 0.33 pN m2 which is similar to that of the stratum corneum (outermost layer of human skin). The modification of the bending stress was achieved by thickness reduction of the sensor where the 3 um thin sensor had two orders of magnitude higher than the sub-300 nm sensor. Moreover, due to conformal contact of thin electrodes, the sensor showed motion artifact-less monitoring up to skin vibration of 15 μm. Sub-300 nm sensor had the highest peel strength (135.09 mN cm^−1^) compared to various thicknesses of sensors, where the trend shows a decrease in peel strength as the thickness increases. Moreover, wrinkle periodicity, wrinkle height, and bending stiffness significantly decreased as the thickness decreased. Due to the conformal contact of the electrode enhanced by its ultrathin structure, the sensor showed SNR of 21.09 dB for ECG and 30.08 dB for EMG, while the Ag/AgCl electrode showed 21.04 and 30.04, respectively, proving the effectiveness of ultrathin structure as high performance soft–dry electrode. On the other hand, an ultrathin tattoo was highlighted as an electrode that could achieve conformal contact with the skin [114,115]. Ameri et al. [115] demonstrated a different form of the electrode to achieve ultrathin and conformal structure by proposing an ultrathin graphene tattoo electrode (GET) (Figure 7e) that can fully conform to the rough skin surface without using the additional adhesives. This conformability improved the SNR by increasing the contact area of the skin to electrode interface and hence lowering the skin to electrode impedance. Using the graphene as base material, to overcome the limitation of graphene that it is too thick to be fully conformable, GET sensors have used wet transfer and dry patterning fabrication process and made ultra-thin GET sensors with a total thickness of 463 ± 30 nm. GET was able to conformally adhere to the skin only with van der Waals interaction and the conformal contact was kept even with the arbitrary skin deformation. The GET sensor had electrode-skin impedance was comparable to the gel electrode and the impedance of the GET sensor was 1 order of magnitude smaller than the Gr/PI sensor that has 12 μm. Moreover, the SNR of the GEPS (electrophysiological sensor using GET) was 15.22 dB while the gel electrode showed 11 dB demonstrating its high performance as a soft–dry electrode.

## 4. Breathability

The human body exudes 600–900 g m^−2^ h^−1^ (for adults) of water every day through the skin and carbon hydroxide, and oxygen gas [118]. These liquid and gas discharges from our skin became a cause of discomfort such as irritation or itching when we used a soft–dry electrode as these electrodes did not have good breathability [119]. Moreover, the sweat and gases that are stagnated between our skin and the sensor cause problems such as degrading sensing signal by increasing skin impedance, toxicity due to metal reaction with sweat, delamination of the electrode due to weakened adhesion, and hindering the electrode for robust long-term monitoring [119,120,121,122]. The sweats and gases produce a humid environment susceptible to the multiplication of bacteria [123,124,125]. The next generation of soft–dry electronic devices has been focusing on the breathability of soft–dry electrodes to address these problems. The criterion of these soft–dry electrodes is water vapor transmission rate (WVTR, g m^−2^ day^−1^) which measures the amount of water vapor being transmitted through a target with restricted temperature and humidity. Each technique offers separate capabilities for fabrication complexity, cost, breathability, etc. This section will categorize recent advances in breathable soft–dry electronics into three types. Each type represents how the individual research pursued breathability to their soft electronics and be discussed in each chapter, some of which are summarized in Table 7.

**Figure 8:**
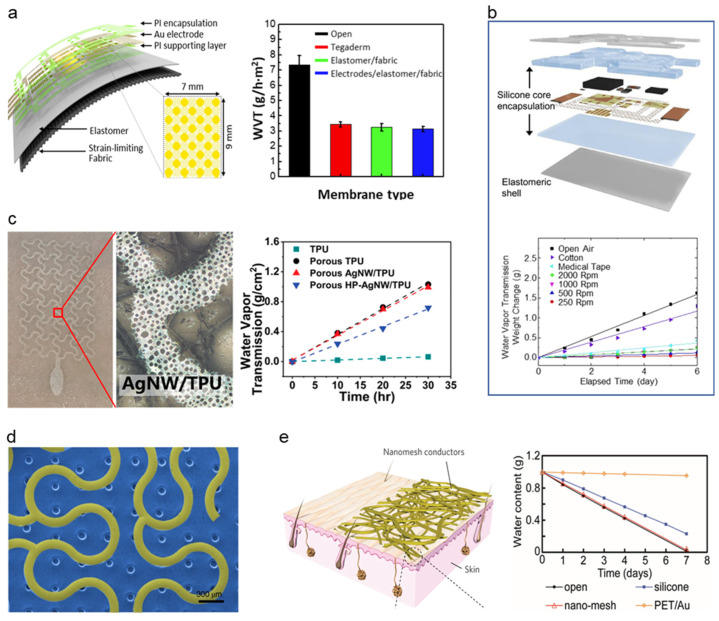
Types of breathability in various soft electronics. (**a**) Breathable elastomer/fabric used electronics. (Reprinted with permission from *Biosens. Bioelectron.* 2020, *165*, 112404, Copyright 2021 Elsevier) [126]. (**b**) Breathable sensor with silicone/elastomeric encapsulation. (Reprinted with permission from *Sci. Adv.* 2016, *2*, e1601185, Copyright 2016 Science Advanced) [127]. (**c**) Gas-permeable porous electrodes. (Reprinted with permission from *ACS Nano* 2020, *14*, 5, 5798–5805, Copyright 2020 ACS) [119]. (**d**) Breathable electrode with a microperforated soft silicone layer. (Reprinted with permission from *Nat. Biomed. Eng.* 2019, *3*, 194–205, Copyright 2019 Nature communications) [129]. (**e**) Substrate-free Nano mesh conductors. (Reprinted with permission from *Nat. Nanotechnol.* 2017, *12*, 907–913, Copyright 2017 Nature Nanotechnology) [24].

The attempt to adopt breathability in the soft–dry electrode started with using intrinsically porous elastomer to adopt breathability. Kwon et al. [126] have proposed a breathable, large-area epidermal electronics system (L-EES) allowing long-term EMG recording without epidermis disruption [131], shown in Figure 8a, by integrating soft breathable elastomer and an array of electrodes. Soft and breathable elastomer is fabricated with elastomer (Ecoflex) and fabric (48% polyamide, 36% polyester, and 16% lycra). The breathable elastomer and fabric composite with electrodes showed decent breathability of 3.13 ± 0.18 g h^−1^ m^−2^ compared to that of commonly used Tegaderm (3.43 ± 0.18 g h^−1^ m^−2^), L-EES showed long-term wearability with a minimal temperature change no rash. The performance of soft–dry electrodes was proven by minimal reduction of signal-to-noise ratio (SNR) (from stationary measuring to measuring in walking conditions) of ~9 dB to ~8 dB, whereas the gel type electrode showed ~9 dB to ~4 dB. Using intrinsically breathable elastomers and fabrics, Jang et al. [121] demonstrated breathable and stretchable electronics for transcutaneous monitoring, shown in Figure 8b, by using intrinsically breathable ultralow modulus silicone (UL-Sil) (Silbione RT Get 47 14 A/B, Bluestar Silicones, USA) with breathable elastic fabric (90% nylon and 10% spandex) to compose a breathable substrate. The breathability of the Silbione was proved by a comparison test with four common silicone elastomers (PDMS, Ecoflex, Solaris), where Silbione showed a transepidermal water loss of ~9 g h^−1^ m^−2^. In contrast, other silicone elastomers showed less than 2 g h^−1^ m^−2^. With the adaptation of a core/shell encapsulation layer, Liu et al. [127] demonstrated breathable epidermal mechano-acoustic sensing electronics (Figure 8b). The WVTR of the core/shell encapsulation layer was 2 mg h^−1^, while the commercialized medical dressings showed 3 mg h^−1^ proving that the WVTR of the core/shell encapsulation layer is comparable to that of the widely used medical tape. The electrode with Silbione and fabric composite elastomer featured a breathable and conformable surface for long-term monitoring, efficient adhesion resulting in minimal restraint on the subject’s motion and providing feasible for robust biopotential recordings such as EMG, ECG, and EOG.

However, due to insufficient breathability of intrinsically breathable material compared to human perspiration rate, more recent studies have tried to pursue higher breathability by post-processing the porous/nonporous materials using a method such as sugar templating, bubble blowing, phase separation, etc. Xu et al. [128] not only increased the breathability of the substrate by multiscale pore system of polystyrene-block-poly(ethylene-ran-butylene)-block-polystyrene (SEBS) substrates but also introduced a self-cooling system by backscattering sunlight and not reflecting human-body mid-infrared radiation to absorb heat dissipation. Using the unstable characteristic of chloroform, the multiscale porous structure was fabricated by mixing IPA and SEBS in chloroform (solvent) and activating the phase separation of IPA from SEBS by rabid evaporation of chloroform, forming Nano/microscale IPA droplets. Then, as the IPA droplets evaporate, the pores are formed in the SEBS substrate, adding ~70% porosity and ~100 um thickness. Multiscale pores provided effective sweat evaporation and gas/vapor diffusion. The WVTR of this substrate was up to 20.6 mg cm^−2^ h^−1,^ which is 69 times higher than the WVTR value of unprocessed SEBS and comparable with nanoPE fabric (23 mg cm^−2^ h^−1^). The breathable SEBS substrate-based electrode showed stable long-term and continuous ECGs recording up to 24 h. Multiscale pore system of SEBS substrate demonstrated promise in long-term comfort and stable ECGs measurement along with passive cooling ability.

PDMS, polyethylene terephthalate (PET), and polyimide (PI) are commonly used as substrates for the epidermal device. However, these materials lack breathability. In need of an easy fabrication process, long-term stability, and breathable and ultrathin material, Zhou et al. [119] proposed a breathable HP-AgNW/TPU film by assembling porous substrates using the breath figure method. The breath figure method is a method to produce breath figure array film (Figure 8c). By constantly dropping water droplets onto substrate solution, the droplet array in the substrate eventually evaporates, leaving the array of breath figure [132]. The HP-AgNW/TPU film showed a WVTR of ~0.023 g cm^−2^ h^−1^, while the untreated TPU film showed a WVTR of less than ~0.002 g cm^−2^ h^−1^. The pore size generated by the breath figure method could be scaled by differing the solution concentration. The optimized concentration was 1.5 wt% TPU and 0.15 wt% PEG. Higher concentration (2 wt% TPU and 0.2 wt% PEG) resulted in a higher chance of getting a dead-end pore. The lower concentration resulted in too large pore size, limiting the precise patterning of the electrode. The HP-AgNW/TPU film with optimized concentration showed a pore diameter of ~40 μm, and the pore covered 39% of the film. The excellent breathability of HP-AgNW/TPU film enhanced its long-term wearability that seven days of use on actual human skin reported neither any allergic reactions nor sweat accumulation, proving the comfort and durability of breathable electronics. The SNR of the ECG measured by the AgNW/TPU film (7.0 dB) was comparable to that of the Ag/AgCl electrode (7.1 dB), and the SNR of the EMG measured with AgNW/TPU film (24.9 dB) was also comparable with that of Ag/AgCl electrode (25.9 dB). While the commercial electrode uses a conductive gel that could irritate skin and cause signal weakening due to dehydration of the gel, AgNW/TPU showed feasible for long-term stability, user comfort due to its breathability, and high SNR without the usage of the conductive gel proving its high performance as a soft–dry electrode.

The electrode with a personalized breathability system according to user skin condition was able to be achieved with micro-perforation of a soft silicone layer. Tian et al. [129] demonstrated a large-area MRI-compatible epidermal electrode array for prosthetic control and cognitive monitoring. The breathability was introduced to the electrode by adopting micropores to the adhesive silicone layer (RT GEL 4642, Blue star) shown in Figure 8d. The pores were micro perforated on the soft silicon layer by inserting a layer of poly (methyl methacrylate) microspheres with a diameter of 100 μm onto the surface of the silicone layer, thermal cure it, and dissolving poly (methyl methacrylate) by soaking in acetone. It was reported that the user’s skin condition determines how much water must be transmitted. Therefore, personalized breathability was enabled by varying the size of the microspheres. The electrode was composed of a microperforated silicone layer and the external-facing layer (Ecoflex) that protects the electrode from the surrounding environment. The WVTR of the electrode was inversely proportional to the thickness of the external layer and directly proportional to the pore size/pore density of the silicone adhesive layer. The 12% microperforated silicone adhesive layer (pore opening diameter of 80 μm and density of 200 pores per cm^−2^) with 8 μm of external layer showed twice higher WVTR of PDMS with the same thickness. Moreover, the breathability achievable with the microperforated silicone design was ~85% of open evaporation condition and was comparable to mesh structured electrodes with high breathability [24]. The large-area epidermal electrode shows promise in long-term EEG monitoring by five-day continuous measurement along with normal routines such as showering, exercising, etc.

Like fabricating simple and effective on-skin electrodes with high breathability, Sun et al. [58] reported the gas-permeable, multifunctional on-skin electronics using laser-patterned porous graphene electrodes and sugar-templated elastomer sponges as breathable substrate. The sugar-templated elastomer sponge was fabricated in a simple and effective approach by making an elastomer/sugar composite and dissolving the sugar is cured composite resulting in an elastomer sponge with effective breathability. Such elastomer sponge with porous graphene showed WVTR of 18 mg cm^−2^ h^−1^ while the elastomer substrates without pore showed WVTR of 1 mg cm^−2^ h^−1^. Enhanced breathability helps sensors to be more durable. The sugar-templated graphene electrode showed long-term feasibility with no inflammation for 7 days of attachment from the volunteers, while the nonporous elastomer substrate gained slight inflammation and was tested by volunteers. With the blind test of the same two elastomers (sugar-templated and nonporous elastomer), nonporous silicone gained a discomfort rate of 3.8 (0 to 10 scale), while the sugar templated graphene sensor gained a discomfort rate of 1.1 showing high comfort through its breathability. The porous structure of this sensor demonstrated high water vapor transmission along with positive feedback for user comfort.

One other approach to overcome the low breathability of the conventional plastic and elastomer film and to fabricate a breathable health monitoring device for inflammation-free, user comfort, and long-term measurement, is the concept of substrate-free on-skin electronics. Substrate-free electronics often take the structure of lightweight open mesh that could be directly imported to the human skin. Miyamoto et al. [24] demonstrated the inflammation-free, gas-permeable, lightweight, stretchable on-skin electronics based on an open nanomesh structure for minimal invasive touch, temperature, and pressure measuring wearable device. After preparing 300–500 nm electrospun PVA nanofiber in a mesh-like form, a 70–100 nm-thick Au layer was deposited on top, forming a mesh-like conductor that could adhere to human skin. After the mesh was deposited on the skin, water was sprayed, dissolving PVA nanofibers and attaching the skin’s nanomesh conductor. (Figure 8e). The SEM image of the Au nanomesh conductor showed that the conductor was attached around sweat pore in human skin without disturbing sweat secretion showing its excellent breathability and thus resulting in a notable reduction of skin irritation and inflammation. For the water transmission testing, the nanomesh conductor reported the same transmission rate as an open bottle demonstrating excellent water permeability of the electrode. Due to the high breathability, the nanomesh conductor gave comfort attachment to the users. The survey of the test participant proved the comfortability of Au nanomesh conductor by showing a 1.16 discomfort rate of nanomesh conductor while 1.83 and 3.39 discomfort ratings for parylene film and silicon film on a scale of 0–10. Moreover, one week of on-skin testing resulted in a significantly low inflammation rate except for one subject with metal allergy showing feasible for wireless touch, temperature, pressure, and EMG with excellent comfort and durability.

While substrate-free mesh-type electrodes showed excellent breathability, hydrophilic treatment to the substrate-free electrodes significantly increased the breathability of the electrode. The contact angle of the water droplet to the skin affects the evaporation rate. When the contact angle of the liquid droplet decreases, the droplet gets thinner, increases the contact area to the surface, and gets thinner, resulting in higher heat conduction that goes through the droplet [133]. Wang et al. [130] reported epidermal electrodes with enhanced breathability and high sensing performance by increasing the hydrophilicity of the skin due to hydrophilic treatment on the Au nanomeshes (AuNM). The hydrophilic treatment was operated by compiling 1-thioglycerol molecules with an AuNM electrode. The water contact angle of the skin is ~75°, and it is not enough to fully wet the skin. However, with the hydrophilic treatment, the contact angle decreased to 10° showing great breathability. The hydrophilic treatment of AuNM took 3.7 s for the sweat evaporation while the bare skin took 13.7 s and the untreated AuNM took ~6.3 s, proving the excellence of hydrophilic treatment in improving sweat evaporation. The treated AuNM showed stable resistance on 6 cycles of sweating and evaporating for 6 h. After being submerged in water for 40 min, it showed stable resistance and was washed with running water for 2 min. There was no report of skin damage after being contacted for five days. Treated AuNM functioned as a joule heater generating an appropriate temperature of 40–60 °C due to its breathable, flexible, and conformable features. Overall, treated AuNM showed superior breathability compared to most of the electrodes in terms of sweat evaporation and could be usable in hot and humid weather and during the activity that accelerates perspiration for long-term and comfortable measurement.

## 5. Outlook

Noble metals, including Au, Ti, and Pt, have also been popular dry electrode materials attributed to high oxidation resistance and good conductivity. But limitations are in scalable manufacturing due to high material costs. Future works may include improvements to cost-effective metals such as Ag and Cu with good electrical properties by surface treatment to inhibit the formation of oxidation layers, as demonstrated in recently studied Cu-Ag compound materials which were able to conductivity while showing ultrahigh oxidation resistance [134]. All carbon materials included in this review consist of 2D carbons, including CNT, graphene, and CB. According to material nanostructure characteristics, 2D layered materials distinguish themselves from bulk form due to the extremely high density of active surface sites, which offer a large contact area, making them suitable for biosensing applications that require compact form factors while maintaining formality and conductivity [135,136]. However, the cytotoxicity of CNT remains primarily debated, and discussions vary between studies due to oxidative stress from reacting with various surrounding materials [137]. Future studies comparing cytotoxicity to substrate types may provide further insight into the credibility of CNT as a stable biomaterial. Conductive polymers such as PEDOT:PSS and PPy are highly favorable dry electrode materials due to their excellent electrical properties and transparency. The proposed polymer-based dry electrodes feature mechanical compliance and ultra-low thickness, proving feasible for robust biosignal recording as low-cost wearable electronics for vital signs and cardiovascular monitoring. Overall, recent studies demonstrate the usage of multiple conductive, flexible, biocompatible material types for improved mechanical and electroconductive stability of the dry electrode. Combining more than one metal, carbon, or polymer nanomaterials has improved material properties such as nanoparticle conductivity, as demonstrated in AgNW-CNT interconnects [77]. A combination of polymer and carbon nanomaterials may propose a solution to scalability by reducing the mass ratio of high-cost carbon ($85,000–300,000 per metric ton) [138] while maintaining conductivity through polymer linkage. The high biopotential measuring capability of novel nanomaterials such as MXene also enlightens future research exploring novel conductive nanomaterials for biosensing applications. Stable signal acquisition capability, reusability, and wearability are essential features that soft–dry electrodes collecting EP signals should have. The use of a nature-inspired structure on dry adhesive enabled excellent adhesion without the issue of skin damage during long-term wear due to its unique and biocompatible design. Adopting adhesion from the elastomer by mixing additive chemicals, giving adhesion directly to conductive elastomer by the same method, and using nature-inspired material such as dopamine that mussel uses has been a popular method fabricate PSA. Lowering Young’s modulus of the electrode and fabricating the ultrathin electrode showed promise in conformal contact of the skin–electrode interface. Chemicals, such as Triton X or glycerol, helped reduce young’s modulus of the elastomer helping its conformal contact. Moreover, the ultrathin electrode and tattoo electrode in nanoscale showed great conformity. There have been various approaches to improve the air permeability of the electrode for the empirical use of the wearable EP monitoring device. Recent breathable electrodes highlight the high WVTR that could reduce skin inflammation issues by not leaving any residue and long-term feasibility resulting from the maintained adhesion and clean contact area of the skin–electrode interface.

## 6. Conclusions

This review summarizes the essential requirements to develop high-performance soft electrodes. Recent advances in several nano-micromanufacturing technologies include direct writing, patterning, and coating. Furthermore, various materials, such as metals, carbon, conductive polymers, and composites have been reported to provide a foundation for developing better dry electrodes. We discuss novel methods to improve physical adhesion, including adhesive and conformal contact of dry electrodes. Furthermore, breathable electrodes for better wearability and stability in long-term measurements are emphasized. Each study suggests solutions to overcome the limitations of dry electrodes while showing their possibility as a high-performance electrodes. Soft and dry electrodes with excellent sensing performance and wearability have been successfully proven in various applications by integrating circuits and wireless communication modules. Beyond device manufacturing and performance optimization, multidisciplinary convergence research has been successfully conducted, including clinical studies, machine-learning approaches, applied research to disease diagnosis, health monitoring, gesture recognition, prosthetic control, and emotional state detection. However, continuous developments are still required for commercialization and industrialization, such as process and material synthesis optimization, SW system development, and system integration. With such enhancements, we believe that the high-performance soft electrodes will be more practical to be used in diverse wearable health monitors and electronics systems.

## Figures and Tables

**Table 2:** Summary of carbon-based soft–dry electrodes.

Electrode Material	Manufacturing Method	Conductivity/ Resistivity	SNR/Contact Impedance	Stretchability	Thickness	Ref.
Silk sericin-CNT ink	Sericin extraction, Drop casting	42.1 ± 1.8 S cm^−1^	N/A	N/A	N/A	[55]
Functionalized conductive graphene (FCG)	AJP, Spin-coating, Photonic-sintering	1.15 Ω	9.5 dB SNR	60%	3.1 nm	[56]
CNT encapsulation onto textile surface	Laser patterning, Heat lamination	N/A	3.4 × 10^4^~ 1.4 × 10^7^ Ω (@ 100 Hz)	20–30%	5–10 μm	[57]
Laser-induced porous graphene (LIG)	Direct laser patterning	10.96 Ω/sq	~17 kΩ (@ 100 Hz)	60%	~20 μm	[58]
PDMS-CB	Casting, Thin film lamination to curved surface	1 × 10^−10^–1 × 10^−3^ S/m	13–18 kΩ (@ 10–10,000 Hz)	N/A	30–100 μm	[59]

**Table 3:** Summary of conductive polymer-based soft–dry electrodes.

Electrode Material	Manufacturing Method	Conductivity/ Resistivity	SNR/Contact Impedance	Stretchability	Thickness	Ref.
PEDOT:PSS/PEO	IJP	84 Ω	N/A	<50%	0.5–4 μm	[70]
PEDOT:PSS	Glyceroand andpolysorbate additive treatment	70–140 S/cm	7 × 10^2^–3 × 10^5^ Ω/cm^2^ (@ 10–10^5^ Hz)	90%	20 μm	[71]
PEDOT:PSS:DVS coated textile	Drop casting	605.9 ± 28.4 S cm^−1^	1.5 × 10^2^–1 × 10^5^ Ω (@ 0.1–10^5^ Hz)	<15% ± 0.4%	100 nm	[72]
PEDOT:PSS, Waterborne polyurethane, D-sorbitol	Casting blended solution to mold	100–600 S/cm	82 kΩ cm^2^ (@ 10 Hz)	43%	20 μm	[73]
Self-adhesive conductive polymer (SACP)	Supermolecular solvent-doping	1–37 S/cm	N/A	700%	<150 μm	[74]
Conductive polymer polypyrrole (PPy)	In-situ polymerization	18.5–24.7 Ω cm^−2^ (@ 1 Hz)	28 Ω cm^−2^	N/A	N/A	[75]

**Table 5:** Summary of various adhesives for dry electrodes.

Type	Material	Structure	Adhesion Strength	Adhesive Durability	Other Characteristics	Ref.
Dry adhesive	Carbon Nanocomposite	Micropillar	~1.3 N cm^−2^	30 attaching cycles	Underwater usable	[92]
	Vinyl siloxane-PDMS	microfibrillar film	1.8 N cm^−2^	Bending, 300 cycles	Rough skin usable	[93]
	Graphene Coated fabric	Octopus-like patterned structure	1.89 N cm^−2^	N/A	Wet skin usable	[94]
	Ag/AgCl, TPU	Fructus Xanthii mimic structure	N/A	Bending, 5000 cycles	Hairy skin usable	[95]
PSA	PDMS/PEIE/CNT composite	Thin film	3 N cm^−2^	100 attaching cycles	Usable under sweat condition	[96]
	PWS	Thin film	0.41 N/cm	N/A	High resistance to motion artifact	[73]
	DMPA-DA	Adhesive protein	~6.2 N cm^−2^	N/A	Sweat resistant	[97]

**Table 6:** Summary of various conformal contact types for soft–dry electrodes.

Type	Material	Structure	Thickness	Young’s Modulus	Adhesion Force	SNR/Contact Impedance	Stretchability	Ref.
Material modification	PEDOT:PSS, glycerol-silk fiber mat	Silk fiber mat structure	20–30 μm	<3 MPa	N/A	~90 kΩ (at 1 kHz)	~250%	[112]
	PEDOT:PSS, glycerol, polysorbate	Fibrous structure	20 μm	0.8 MPa	~1.2 N/cm	35.23 dB	90%	[71]
	AgNW, PDMS, Triton X	a-PDMS matrix	N/A	40 ± 5 kPa	~25 N/m	~20 kΩ (at 1 kHz)	400%	[113]
Structure modification	Au, parylene	Thin film structure	300 nm	20–150 kPa	N/A	44 kΩ (at 1 kHz)/ 21.09 dB	60%	[111]
	PEDOT:PSS, graphene, SDS, BSL	Thin film structure	100 nm	640 kPa	N/A	32 kΩ (at 100 Hz)/ 23 ± 0.7 dB	40%	[80]
	PEDOT:PSS, Au	Tattoo structure	600–1200 nm	1 GPa	N/A	294 kΩ (at 60 Hz, after 60 min)	10%	[114]
	Graphene, PMMA	Tattoo structure	463 nm	20–150 kPa	N/A	15.22 dB	40%	[115]

**Table 7:** Summary of performance and key characteristics of breathable soft electrodes.

Type	Material	Core Breathable Characteristics	Thickness	Breathability (WVTR ^a^)	Ref.
Intrinsically breathable material substrates	Au, PI, elastomer, fabric	Composite of breathable elastomer and the fabric	~1.1 mm	0.31 mg cm^−2^ h^−1^	[126]
	Cellular silicone/elastomeric fabric	Composite of breathable elastomer and the fabric	Elastomer (~100 μm), fabric (~1 mm)	~1 mg cm^−2^ h^−1^	[121]
	Silbione, Ecoflex	Composite of breathable silicone and elastomer layer	2 mm	2 mg h^−1^	[127]
Post-processed Porous substrates	SEBS	Phase separation generated pores	~100 μm	20.6 mg cm^−2^ h^−1^	[128]
	AgNW, TPU	Breathable pores generated with breath figure method	4.6 μm	23 mg cm^−2^ h^−1^	[119]
	Silbione/Ecoflex	Microperforated silicone layer	88 μm	can be controlled	[129]
	Graphene, silicone elastomer sponge	Sugar templated porous silicone elastomer sponges	500 μm	18 mg cm^−2^ h^−1^	[58]
Substrate-free	Au Nanomesh	Breathable Nano mesh directly attached on skin	Several tens of nanometers	5.95 mg h^−1^	[24]
	Au Nanomesh	Hydrophilically treated breathable Au nanomeshes design	30 nm	N/A	[130]

^a^ WVTR, Water vapor transmission rate.

## Data Availability

No new data were created or analyzed in this study.
